# Global, regional and national consumption of major food groups in 1990 and 2010: a systematic analysis including 266 country-specific nutrition surveys worldwide

**DOI:** 10.1136/bmjopen-2015-008705

**Published:** 2015-09-24

**Authors:** Renata Micha, Shahab Khatibzadeh, Peilin Shi, Kathryn G Andrews, Rebecca E Engell, Dariush Mozaffarian

**Affiliations:** 1 Friedman School of Nutrition Science and Policy, Tufts University, Boston, Massachusetts, USA; 2 Department of Epidemiology, Harvard School of Public Health, Boston, Massachusetts, USA; 3 Institute of Health Metrics and Evaluation, Seattle, Washington, USA

**Keywords:** EPIDEMIOLOGY, PUBLIC HEALTH

## Abstract

**Objective:**

To quantify global intakes of key foods related to non-communicable diseases in adults by region (n=21), country (n=187), age and sex, in 1990 and 2010.

**Design:**

We searched and obtained individual-level intake data in 16 age/sex groups worldwide from 266 surveys across 113 countries. We combined these data with food balance sheets available in all nations and years. A hierarchical Bayesian model estimated mean food intake and associated uncertainty for each age-sex-country-year stratum, accounting for differences in intakes versus availability, survey methods and representativeness, and sampling and modelling uncertainty.

**Setting/population:**

Global adult population, by age, sex, country and time.

**Results:**

In 2010, global fruit intake was 81.3 g/day (95% uncertainty interval 78.9–83.7), with country-specific intakes ranging from 19.2–325.1 g/day; in only 2 countries (representing 0.4% of the world's population), mean intakes met recommended targets of ≥300 g/day. Country-specific vegetable intake ranged from 34.6–493.1 g/day (global mean=208.8 g/day); corresponding values for nuts/seeds were 0.2–152.7 g/day (8.9 g/day); for whole grains, 1.3–334.3 g/day (38.4 g/day); for seafood, 6.0–87.6 g/day (27.9 g/day); for red meats, 3.0–124.2 g/day (41.8 g/day); and for processed meats, 2.5–66.1 g/day (13.7 g/day). Mean national intakes met recommended targets in countries representing 0.4% of the global population for vegetables (≥400 g/day); 9.6% for nuts/seeds (≥4 (28.35 g) servings/week); 7.6% for whole grains (≥2.5 (50 g) servings/day); 4.4% for seafood (≥3.5 (100 g) servings/week); 20.3% for red meats (≤1 (100 g) serving/week); and 38.5% for processed meats (≤1 (50 g) serving/week). Intakes of healthful foods were generally higher and of less healthful foods generally lower at older ages. Intakes were generally similar by sex. Vegetable, seafood and processed meat intakes were stable over time; fruits, nuts/seeds and red meat, increased; and whole grains, decreased.

**Conclusions:**

These global dietary data by nation, age and sex identify key challenges and opportunities for optimising diets, informing policies and priorities for improving global health.

**Strengths and limitations of this study**

- Suboptimal diet is now the leading risk factor for non-communicable diseases; intakes of specific foods, rather than macronutrients or micronutrients, may be most relevant for non-communicable disease risk. This is the first study to provide comprehensive and comparable quantitative estimates, based on individual-level global intakes and their uncertainties, of key foods influencing chronic diseases, including by region, country, age, sex and time.
- We identified and made use of a much larger set of primary data sources than previous collations, which have relied mainly on crude availability or expenditure data that may not accurately reflect individual intake.
- These global data identify key challenges and opportunities for optimising diets; facilitate quantification of disease burdens attributable to key foods; and inform policies and priorities for improving global health.
- Primary data were deficient for certain foods (nuts/seeds, whole grains), countries (particularly in the Sub-Saharan African regions) and the 1990 time period. Our investigation takes advantage of the Food and Agriculture Organization of the United Nations (FAO) food balance sheet data in a multilevel Bayesian model, to provide additional information across all countries and years, but to also be appropriately adjusted to account for their error and variation based on relationships with multiple individual dietary surveys in countries having both. The model further accounted for differences in survey methods and representativeness, and sampling and modelling uncertainty.
- We focused on foods with probable or convincing evidence for impact on chronic diseases, and many other foods were not included. Thus, our findings represent the best available, yet still imperfect, data on current global dietary intakes of key foods, which are updated and expanded on in ongoing work (http://www.globaldietarydatabase.org; anticipated results in 2018).

## Introduction

Non-communicable diseases (NCDs), including cardiovascular diseases (CVDs), cancer and diabetes mellitus, are the leading cause of mortality and disability worldwide.^1^ NCD burdens are expected to further increase with population ageing, and increasing rates of obesity and other diet-related risk factors. Suboptimal diet is now the leading risk factor for NCDs.^2^ ^3^ Even modest dietary changes are associated with meaningful reductions in CVD morbidity and mortality, type 2 diabetes, specific cancer sites and major risk factors including hypercholesterolaemia, hypertension and obesity.^4–10^ A growing body of evidence indicates that intakes of specific foods, rather than macronutrients or micronutrients, are most relevant for NCD risk.^7^ ^11^ ^12^ Indeed, among nutritional risk factors for NCDs, nearly all of the top factors globally are foods, not nutrients.^2^ Food-based guidance also greatly facilitates public education and communication efforts.^13–16^ Thus, a better understanding of patterns and distributions of major foods around the world is crucial to establish priorities for dietary guidelines and to inform, design and implement strategies for reducing national and global diet-related diseases.^2^ ^17^

Remarkably, the patterns and distributions of intakes of key foods around the world have not been well established. Most previous global analyses have relied on crude national estimates of food balance sheets from the Food and Agriculture Organization of the United Nations (FAO),^18–21^ or expenditure data (eg, household budget surveys).^22^ Availability and expenditure estimates may not accurately reflect individual dietary intakes; moreover, such national estimates cannot elucidate key differences within populations, such as by age or sex. In addition, prior global dietary estimates have not formally incorporated methodological heterogeneity or statistical uncertainty.

To address these prior limitations and provide the most robust available evidence on dietary intakes of key foods related to chronic diseases worldwide, we identified specific foods related to cardiometabolic and cancer risks; characterised their optimal consumption levels; systematically collected and evaluated global data on intakes based on both individual-level national surveys and national FAO food balance sheets; and, using comparable and standardised methods, constructed a Global Dietary Database of consumption by world region, country, age, sex and time.

## Methods

### Study design

This work was performed by the Nutrition and Chronic Diseases Expert Group (NutriCoDE) as part of the 2010 Global Burden of Diseases, Injuries and Risk Factors (GBD) Study.^2^ Our methods for identification, access and selection of dietary risk factors and data have been reported.^23–25^ Because we used de-identified national data sets, this research was reviewed by our institutional human participants committee and deemed exempt from human participants research requirements. To generate valid, comparable estimates of consumption of foods around the world, we used consistent methods across regions, countries, age and sex subgroups, and time, to:

- Identify specific foods having chronic disease impact, based on strength of evidence for aetiological effects on coronary heart disease (CHD), stroke, type 2 diabetes or diet-related cancers.
- Systematically search for nationally representative data from 187 nations around the world on individual-level dietary consumption of these foods, including by age and sex, from 1980 to 2010.
- Retrieve these individual-level data, including assessment of quality and representativeness, and maximisation of measurement comparability and consistency; as well as data from FAO food balance sheets categorised to correspond to key foods assessed and available in all 187 nations across all years.
- Estimate consumption levels for each food by region, country, age, sex and time, accounting for differences in food intakes versus availability, survey methods and representativeness, and sampling and modelling uncertainty.
- Characterise optimal consumption levels of each food, based on observed intakes associated with lowest disease risk and observed mean national consumption levels globally, to place the observed intakes in context and enable quantification of relevant attributable disease burdens.

### Identification of key foods having an impact on chronic diseases

Our methods for identifying the key foods of interest have been previously reported.^23^ ^26–29^ ^30^ Briefly, we required convincing or probable evidence for aetiological effects on clinical chronic diseases including CHD, stroke, type 2 diabetes and cancers, supported by (but not solely based on) effects on physiological risk factors (eg, blood pressure, obesity, blood cholesterol). We identified evidence for effects of fruits, vegetables, nuts/seeds, whole grains, seafood and processed meats on CHD;^31–36^ of fruits, vegetables and seafood on stroke;^31^ ^37^ of nuts/seeds, whole grains, unprocessed red meats and processed meats on diabetes;^32^ ^38^ ^39^ and of fruits, vegetables, unprocessed red meats and processed meats on cancer.^27^ ^29^

### Systematic searches for national dietary data

**Figure 1:**
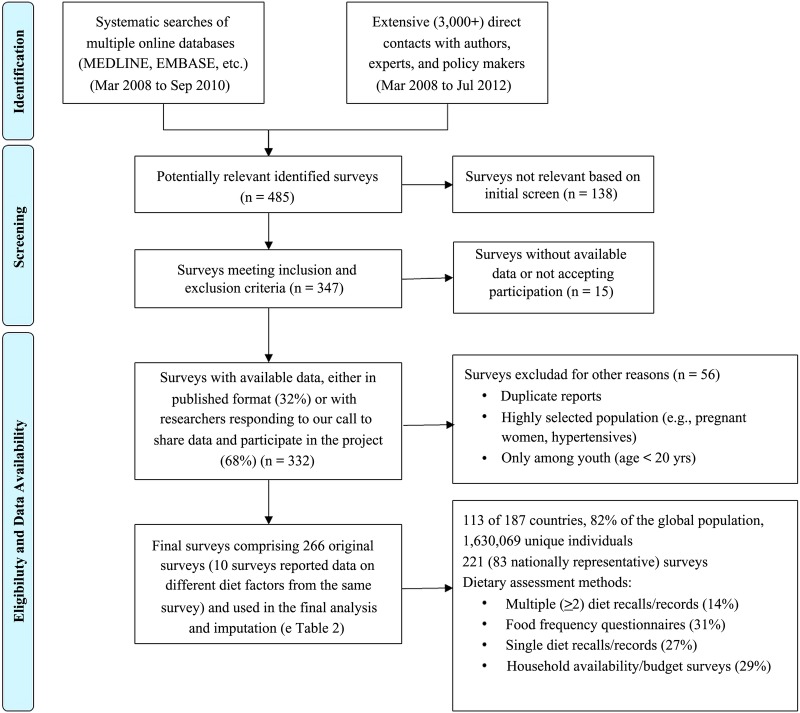
Flow diagram describing the systematic search for nationally representative surveys of food and nutrient intake.

We performed systematic searches for individual-level dietary surveys in all countries, focusing on adults, given our emphasis on chronic diseases. Using standardised criteria and methods that have been described in detail,^23–25^ multiple online databases were searched from March 2008 to September 2010, without date or language restrictions. These searches were further complemented by extensive personal communications with experts and authorities around the world, and by other potential data sources such as large local cohorts, the WHO STEPwise approach to surveillance (STEPS) database and household-level surveys. The results of our search strategy by dietary factor, time and region have been reported.^40^ A total of 266 surveys (83% nationally representative) in adults representing 113 of 187 countries and 82% of the global population were identified (figure 1). We supplemented these data with annual FAO food balance sheet data available in all nations and years.^18^ The FAO food balance sheets capture a country's net annual food availability for human consumption based on reported local production, imports and exports, with adjustment for other uses (livestock and seed).^18^ These estimates were calculated by the FAO as the residual from subtracting utilisation (quantity exported, fed to livestock, used for seed, processed for food use and non-food uses, and losses during storage and transportation) from the total supply (quantity imported and produced, with adjustments for changes in stocks),^18^ ^41^ divided by the population of a given nation.

### Data retrieval and standardisation

Data retrieval followed the 2010 GBD comparative risk assessment framework,^42^ collecting quantitative data on consumption in 16 age-specific and sex-specific subgroups across 21 world regions (see eTable 1) and two time periods (1990 and 2010). The process of evaluating, retrieving, extracting and standardising global data from diet surveys has been published previously.^23–25^ In brief, survey quality was assessed, and optimal and alternative metrics and units were defined for each food. Food consumption was standardised and evaluated as energy-adjusted g/day to 2000 kcal/day using the residual method.^43^ Data sources used to estimate intakes are listed in table 1, and dietary surveys providing data on key foods are listed in eTable 2. FAO individual food items were categorised to correspond to key foods (eg, ‘sunflower seed’, ‘sesame seed’ and ‘treenuts’ corresponded to the nut/seed food group), and subsequently summed to comprise a given food group, for each country and year. We calculated 14 composite diet composition variables specific to the overall 2010 NutriCoDE list of dietary risk factors of interest (with the exception of sodium). The 14 standardised FAO nutrients or food groups represented the majority of food available for human consumption in 187 countries in each year from 1980–2010.^25^

**Table 1:** Data sources, modelling approaches and validation methods used to estimate adult intake levels of key foods worldwide, by region, country, age and sex in 1990 and 2010

Dietary factor	Data sources				Statistical methods used for pooling and modelling data from diverse global sources		
	Individual-level surveys			National FAO food balance sheets†	Modelling approach	Survey-specific covariates‡	Validity
	Regions covered (of 21)*	Years covered	Surveys, countries (of 187) and global population covered				
Fruits	AC, AE, APH, AS, ASE, AUS, CAR, EURC, EURE, EURW, LAC, LAS, LAT, NA, NAM, OC, SSC, SSE, SSS, SSW	1980–2009	A total of 204 surveys, of which 137 had individual-level data (113 of those had age-specific and sex-specific estimates) and 67 were household-level surveys, were collected from 109 countries and represented 87% of the world's adult population	Calculated fruit intake (derived from FAO data on fruits) consumed per capita per day in 187 countries in each year from 1990 to 2010	DisMod3, a Bayesian hierarchical method, was used to pool data from multiple sources and model missing data using informative time-varying covariates, borrowing information across geographical region and time period while also incorporating uncertainty due to measurement error and model specification Models were fit using a randomised Markov chain Monte Carlo algorithm based on the Adaptive Metropolis step function	Metric (primary vs secondary metric), representativeness (nationally representative vs subnational), diet assessment method (diet recalls/records or FFQ vs household availability/budget survey) Country-specific: lag-distributed national per capita income (inflation and purchasing power parity adjusted), FAO factor variables 1, 2, 4	Models were assessed for convergence of Markov chain Monte Carlo iterations. DisMod3 was validated using goodness-of-fit tests and out-of-sample predictive validity tests, in which 10% of data were held out of the model. Qualitative evaluation for foods was conducted by comparing the estimated foods with known high-quality data and assessing their face validity through contact with country experts
Vegetables§	AC, AE, APH, AS, ASE, AUS, CAR, EURC, EURE, EURW, LAC, LAS, LAT, NA, NAM, OC, SSC, SSE, SSS, SSW	1980–2009	A total of 204 surveys, of which 137 had individual-level data (113 of those had age-specific and sex-specific estimates) and 67 were household-level surveys, were collected from 109 countries and represented 87% of the world's adult population	Calculated vegetable intake (derived from FAO data on vegetables) consumed per capita per day in 187 countries in each year from 1990 to 2010		Metric (primary vs secondary metric), representativeness (nationally representative vs subnational), diet assessment method (diet recalls/records or FFQ vs household availability/budget survey)	
Legumes§	AE, APH, AS, ASE, AUS, CAR, EURC, EURE, EURW, LAC, LAS, LAT, NA, NAM, SSE, SSS, SSW		A total of 138 surveys, of which 72 had individual-level data (62 of those had age- and sex-specific estimates) and 66 were household-level surveys, were collected from 64 countries and represented 81% of the world's adult population	Calculated legume intake (derived from FAO data on legumes) consumed per capita per day in 187 countries in each year from 1990 to 2010		Metric (primary vs secondary metric), representativeness (nationally representative vs subnational), diet assessment method (diet recalls/records or FFQ vs household availability/budget survey)	
Nuts and seeds	AE, APH, AS, ASE, AUS, CAR, EURC, EURE, EURW, LAC, LAS, LAT, NA, NAM, SSE, SSS, SSW	1980–2009	A total of 126 surveys, of which 61 had individual-level data (54 of those had age-specific and sex-specific estimates) and 65 were household-level surveys, were collected from 53 countries and represented 74% of the world's adult population	Calculated nut and seed intake (derived from FAO data on nuts/seeds) consumed per capita per day in 187 countries in each year from 1990 to 2010		Representativeness (nationally representative vs subnational), diet assessment method (diet recalls/records or FFQ vs household availability/budget survey)	
Whole grains	AE, APH, ASE, AUS, CAR, EURC, EURW, LAS, LAT, NA, NAM, SSS	1987–2009	A total of 35 surveys, all of which had individual-level data with age-specific and sex-specific estimates, were collected from 25 countries and represented 41% of the world's adult population	Calculated whole grain intake (derived from FAO data on barley, rye and other cereals) consumed per capita per day in 187 countries in each year from 1990 to 2010		Representativeness (nationally representative vs subnational)	
Seafood	AE, APH, AS, ASE, AUS, CAR, EURC, EURE, EURW, LAC, LAT, NA, NAM, SSE, SSS, SSW	1980–2009	A total of 115 surveys, of which 48 had individual-level data (40 of those had age-specific and sex-specific estimates) and 67 were household-level surveys, were collected from 52 countries and represented 54% of the world's adult population	Calculated PUFA n-3 intake (derived from FAO data on PUFA n-3) consumed per capita per day in 187 countries in each year from 1990 to 2010		Representativeness (nationally representative vs subnational)	
Red meats, unprocessed	AC, AE, APH, AS, ASE, AUS, CAR, EURC, EURE, EURW, LAC, LAS, LAT, NA, NAM, SSE, SSS, SSW	1980–2009	A total of 154 surveys, of which 87 had individual-level data (69 of those had age-specific and sex-specific estimates) and 67 were household-level surveys, were collected from 74 countries and represented 83% of the world's adult population	Calculated red meat intake (derived from FAO data on red meats) consumed per capita per day in 187 countries in each year from 1990 to 2010		Metric (primary vs secondary metric), representativeness (nationally representative vs subnational), diet assessment method (diet recalls/records or FFQ vs household availability/budget survey)	
Processed meats	AE, APH, ASE, AUS, CAR, EURC, EURE, EURW, LAC, LAS, LAT, NA, NAM, SSS	1980–2009	A total of 127 surveys, of which 60 had individual-level data (58 of those had age-specific and sex-specific estimates) and 67 were household-level surveys, were collected from 54 countries and represented 54% of the world's adult population	Calculated red meat intake (derived from FAO data on red meats), pig meat intake (derived from FAO data on pig meats) and animal fat intake (derived from FAO data on animal fats) consumed per capita per day in 187 countries in each year from 1990 to 2010		Metric (primary vs secondary metric), representativeness (nationally representative vs subnational), diet assessment method (diet recalls/records or FFQ vs household availability/budget survey)	

*Based on 21 GBD study regions including APH, Asia Pacific, high income; AC, Asia, Central; AE, Asia, East; AS, Asia, South; ASE, Asia, Southeast; AUS, Australasia; CAR, Caribbean; EURC, Europe, Central; EURE, Europe, Eastern; EURW, Europe, Western; LAA, Latin America, Andean; LAC, Latin America, Central; LAS, Latin America, Southern; LAT, Latin America, Tropical; NAM, North Africa/Middle East; NA, North America, high income; OC, Oceania; SSC, Sub-Saharan Africa, Central; SSE, Sub-Saharan Africa, East; SSS, Sub-Saharan Africa, Southern; and SSW, Sub-Saharan Africa, West.

†The FAO food balance sheets capture a country's net annual food availability based on reported local production, imports and exports. We calculated 14 composite diet composition variables from FAO food balance sheets specific to the overall 2010 NutriCoDE list of dietary factors of interest (with the exception of sodium). The 14 standardised FAO nutrients or food groups represented the majority of food available for human consumption in 187 countries in each year from 1990 to 2010.

‡Both survey-specific and national-level covariates were incorporated in the model. Primary inputs were the survey-level intake data and the diet composition variables from FAO food balance sheets, including all available country-specific, time-specific, age-specific and sex-specific consumption levels (mean, distribution), data on the numbers of participants in each strata; and survey-level indicator covariates (sampling representativeness, dietary assessment method, type of dietary metric). Surveys carried out between 1980 and 1997 were used to inform the 1990 time period, and surveys carried out between 1997 and 2010 and the 2010 period. Time-varying country-level covariates (available in all years, including 2010) further informed the estimates, including LDI^44^ (inflation and purchasing power parity adjusted); and national dietary patterns characterised by scores on four factors from a principal component analysis of the 14 FAO diet composition variables.^18^ Taking into account that many of the food covariates are very collinear (eg, red meat, pig meat and animal fats), and that consuming more of one food necessitates consuming less of other types, we used dimension reduction through principal component analysis to reduce the 14 standardised FAO nutrients or food groups into four factor variables, which were included in the model to improve country-level predictions: factor 1 included red meats, animal fats and pig meats; factor 2 included n-3 polyunsaturated fats, n-6 polyunsaturated fats, whole grains, nuts and vegetables; factor 3 included fruits, legumes and nuts; and factor 4 included sugars, stimulants and saturated fats (from oils). The FAO covariates were used in the per cent natural logarithm form, that is, the per cent of total kilocalorie that is comprised of a particular food. A space-time smoothing procedure was used to generate a full time series of intake estimates. Income and education were used as covariates in the space-time model to improve predictions in instances of missing data. For education, the age standardised mean number of years of education for ages 25^+^ by sex as a continuous variable was used.^45^ For income, the estimated and normalised lag-distributed income based on the international dollar as a continuous variable was used.^34^ For countries that had split or merged during the time series (1990–2010), we split/merged these countries into constituent countries using a growth rate method to generate as close to a full time series as possible for all countries. For model description (DisMod3, eAppendix) and model fits (eFigure 7) see data supplements.

§Vegetable and legume intake were estimated separately using Dismod3, and subsequently summed, given that studies evaluating disease risk typically summed or used overlapping categories of vegetable and legumes (eg, green beans included as vegetables).

FAO, Food and Agriculture Organization of the United Nations; FFQ, food frequency questionnaire; GBD, Global Burden of Diseases, Injuries, and Risk Factors; LDL, lag-distributed national per capita income.

### Quantification of global, regional and national distributions

Using systematic data retrieval and standardisation methods, we assessed differences in comparability of individual-level surveys and measures, for example, by representativeness; national, urban or rural coverage; age groups; dietary instruments; and dietary metrics. In addition, whereas FAO food balance sheets utilised comparable methods across nations and years, we recognised that availability data may overestimate true intakes and do not provide information on within-nation heterogeneity, for example, by age or sex.^46^ We thus took advantage of countries having data on both to determine the relationship between individual-level data and the FAO food balance sheet data, accounting for age and sex patterns in the individual-level intakes, to adjust the estimated intakes in those countries having only FAO data.

To account for differences in surveys of intakes versus FAO food balance sheet data, survey methods and representativeness, and sampling and modelling uncertainty, we developed, for each food, an age-integrating Bayesian hierarchical model that estimated the mean intake level and its statistical uncertainty for each age-sex-country-year stratum, as previously described (see table 1 and eAppendix).^2^ ^24^ ^25^ These estimates considered individual-level national survey data as the primary standard, based on the observed relationships between individual-level dietary surveys and FAO food balance sheets in countries that had data on both. By combining both individual-level diet surveys and adjusted FAO data, our dietary estimates in the Global Dietary Database were derived from dietary information available in all 187 countries.

To incorporate and quantify uncertainty, we used the Markov chain Monte Carlo algorithm, based on the Adaptive Metropolis step function analyses, to draw 1000 times from the posterior distribution of each exposure for each age-sex-country-year stratum. We computed the mean exposure from the 1000 draws and the 95% uncertainty interval (UI) as the 2.5th and 97.5th centiles of the 1000 draws, reflecting all key sources of uncertainty (eAppendix). Absolute and relative differences in exposure between 1990 and 2010 were calculated at the draw level to account for the full spectrum of uncertainty.

### Characterisation of optimal consumption

To place observed consumption levels in context and allow separate consideration of potential impact of current intake levels on disease, we characterised, for each food, the optimal, yet feasible, consumption levels to minimise chronic diseases (table 2).^23–25^ ^47^ These were based on (1) the mean observed consumption associated with lower disease risk in meta-analyses of clinical endpoints, (2) the mean national consumption levels actually observed in at least 2–3 countries around the world, and (3) general consistency with major dietary guidelines.

**Table 2:** Optimal consumption levels of key foods related to non-communicable diseases risk*

Foods† (standardised serving size)	Related disease outcomes	Observed consumption levels associated with lowest disease risk in meta-analyses‡	Observed mean national intakes (top or bottom 3 countries) in 2010§	Recommended intakes by major dietary guidelines¶	Optimal population intake (mean±SD)**
Fruits (100 g/serving)	↓ CHD, ↓ stroke, ↓ oesophageal cancer, and ↓ lung cancer	4.4 servings/day (ischaemic stroke) 3.0 servings/day (total stroke) 2.8 servings/day (lung cancer) 2.4 servings/day (CHD) 1.7 servings/day (ESCC) 2.4 servings/day (EAC)	Top 3 countries: Barbados: 418.6 g/day Jamaica: 402.4 g/day Jordan: 302.2 g/day	USDG 2010: 2 cups/day AHA 2020: ≥4.5 cups/day (including vegetables)	300±30 g/day
Vegetables, including legumes (100 g/serving)	↓ CHD, ↓ stroke, ↓ oesophageal cancer	5.3 servings/day (MI) 3.7 servings/day (CHD) 1.5 servings/day (ESCC) 1.8 servings/day (EAC)	Top 3 countries (vegetables): Lebanon: 316.2 g/day China: 305.0 g/day Jordan: 302.3 g/day Top 3 countries (legumes): Brazil: 182.1 g/day Colombia: 126.2 g/day Mexico: 94.5 g/day	USDG 2010: 2½ cups/day (including legumes and starchy vegetables) AHA 2020: ≥4.5 cups/day (including fruits)	400±40 g/day
Nuts/seeds (1 oz (28.35 g)/serving)	↓ CHD, ↓ diabetes	4 times/week (CHD) 4 servings/week (diabetes)	Top 3 countries: Malaysia: 57.2 g/day Lebanon: 30.6 g/day UK: 14.9 g/day	USDG 2010: 4 oz/week (113.4 g/week) (including soy products) AHA 2020: ≥4 (1 oz) servings/week (113.4 g/week) (including legumes)	4 (1 oz=28.35 g)±0.4 servings/week (113.4±11.3 g/week)
Whole grains (50 g/serving)	↓ CHD, ↓ diabetes	2.5 servings/day (CHD) 2.5 servings/day (diabetes)	Top 3 countries: Germany: 149.4 g/day Barbados: 111.7 g/day The Netherlands: 98.3 g/day	USDG 2010: 3 (1 oz) servings/day (85 g/day) AHA 2020: ≥3 (1 oz) servings/day (≥85 g/day)	2.5 (50 g)±0.25 servings/day (100±12.5 g/day)
Seafood (100 g/serving)	↓ CHD, ↓ stroke	3 servings/day (fatal CHD) ≥5 servings/week (total stroke)	Top 3 countries: Japan: 104.2 g/day Iceland: 76.6 g/day South Korea: 73.7 g/day	USDG 2010: 8 oz/week (226.8 g/week) AHA 2020: ≥2 (100 g) servings/week (≥200 g/week)	3.5 (100 g)±0.35 servings/week (350±35 g/week)
Red meats, unprocessed (100 g/serving)	↑ diabetes, ↑ colorectal cancer	0.19 servings/day (diabetes) 0.29 servings/day (colorectal cancer)	Bottom 3 countries: Armenia: 15.0 g/day Georgia: 15.0 g/day Malaysia: 15.8 g/day	USDG 2010: 26 oz/week (737 g/week) (including meat (red and processed), poultry and eggs) AHA 2020: none set	1 (100 g)±0.1 serving/week (100±10 g/week)
Processed meats (50 g/serving)	↑ CHD, ↑ diabetes, ↑ colorectal cancer	0.07 serving/day (CHD) 0.11 serving/day (diabetes) 0.12 serving/day (colorectal cancer)	Bottom 3 countries: South Korea: 3.0 g/day Iran: 3.7 g/day China: 3.9 g/day	USDG 2010: as low as possible AHA 2020: ≤2 (50 g) servings/week (≤100 g/week)	0

*For each dietary factor, the optimal consumption level was identified based both on observed levels at which lowest disease risk occurs and observed mean consumption levels in nations. We also considered whether such identified levels were consistent with major dietary guidelines.^10^ ^48^

†Foods for which we identified probable or convincing evidence for aetiological effects on chronic diseases including CHD, stroke, type 2 diabetes or cancers.^19^ ^23^ For cancers, we based our assessments on the WCRF/AICR report^27^ and subsequent updates.^29^ Based on available evidence, we identified evidence for aetiological effects on CHD of fruits, vegetables, nuts/seeds, whole grains, seafood and processed meats;^31^^–^36 on stroke of fruits, vegetables and seafood;^31^ ^37^ on diabetes of nuts/seeds, whole grains, unprocessed red meats and processed meats;^32^ ^38^ ^39^ and on cancer of fruits, vegetables, unprocessed red meats and processed meats.^27^ ^29^

‡Observed median consumption levels in population subgroups (eg, top or bottom quartile or quintile) associated with lowest disease risk in meta-analyses of prospective cohort studies and/or randomised controlled trials.

§Observed mean national consumption levels in the top (for protective factors) or bottom (for harmful factors) three countries as identified in our global data sources.^24^ ^25^

¶Recommended intake levels based on the USDG 2010 for a 2000 kcal/day diet,^48^ and on the AHA 2020.^10^

**Because not all individuals within a population can have precisely the same exposure level, the plausible distribution (SD) of optimal consumption was calculated from the average SD for all metabolic risk factors in the GBD study (10% of the mean).

AHA 2020, 2020 American Heart Association Impact Goals; CHD, coronary heart disease; EAC, oesophageal adenocarcinoma; ESCC, oesophageal squamous cell carcinoma; GBD, global burden of disease; MI, myocardial infarction; USDG 2010, US Department of Agriculture 2010 Dietary Guidelines for Americans; WCRF/AICR, World Cancer Research Fund/American Institute for Cancer Research.

## Results

### Global consumption in 2010

**Figure 2:**
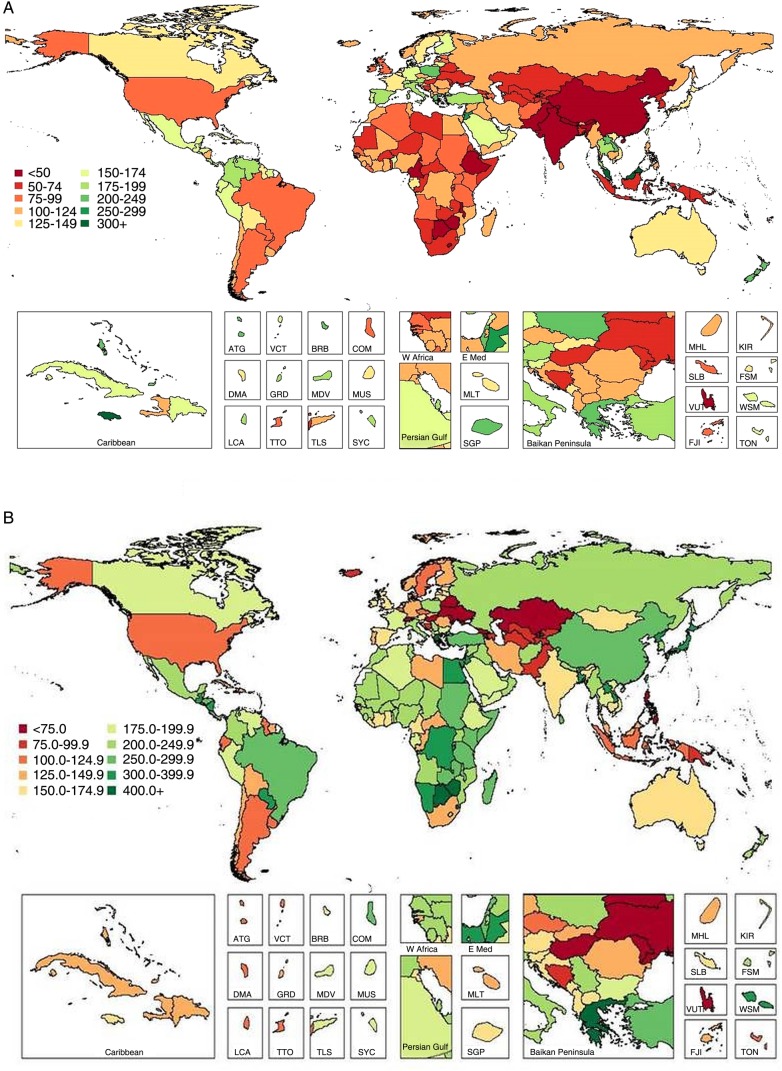
Global and regional mean fruit (A) and vegetables (B) intake (g/d) in 2010 for adults ≥20 years of age in 2010 (see eTable 3 for numerical mean estimates and uncertainty intervals).

In 2010, mean global fruit consumption in adults was 81.3 g/day (95% UI 78.9–83.7; table 3 and figure 2), yet with 6-fold variation across 21 world regions (from 27.6 to 169.9 g/day; figure 5) and 17-fold variation across 187 countries (from 19.2 to 325.1 g/day). Highest intakes were identified in Jamaica, Malaysia, Jordan, Greece and New Zealand (see eTable 3); and lowest intakes in Ethiopia, Nepal, India, Vanuatu and Pakistan. Only 2 of 187 countries, Jamaica and Malaysia, representing roughly 19 million adults (0.4% of the global adult population), had mean consumption ≥300 g/day.

**Table 3:** Characteristics of global consumption of key foods in 2010

Characteristics of global consumption in 2010	Fruits	Vegetables	Nuts/seeds	Whole grains	Seafood	Red meats, unprocessed	Processed meats
Global mean consumption (95% uncertainty interval)	81.3 g/day (78.9–83.7)	208.8 g/day (203.4–214.3)	8.9 g/day (8.3–9.5)	38.4 g/day (35.5–41.7)	27.9 g/day (26.9–29.1)	41.8 g/day (40.8–42.8)	13.7 g/day (13.2–14.3)
Range across 21 global regions (overall variation)	27.6–169.9 g/day (6-fold)	86.1–294.4 g/day (3.4-fold)	0.3–32.6 g/day (117.4-fold)	9.4–144.9 g/day (15.4-fold)	9.2–81.3 g/day (9-fold)	7.3–91.3 g/day (12.5-fold)	4.1–44.4 g/day (11-fold)
Regions with highest levels (mean consumption)	Central Latin America (169.9 g/day), Australasia (166.2 g/day), Western Europe (165.2 g/day), Caribbean (165.0 g/day), Andean Latin America (148.5 g/day)	East Asia (294.4 g/day), high-income Asia Pacific (281.5 g/day), Central Sub-Saharan Africa (273.9 g/day), Tropical Latin America (260.4 g/day), East Sub-Saharan Africa (243.0 g/day)	Southeast Asia (32.6 g/day), West Sub-Saharan Africa (16.3 g/day), Eastern Europe (11.2), North Africa/Middle East (10.9 g/day), South Asia (10.9 g/day)	Southeast Asia (144.9 g/day), Southern Sub-Saharan Africa (111.5 g/day), East Sub-Saharan Africa (74.5 g/day), West Sub-Saharan Africa (74.0 g/day), Australasia (71.6 g/day)	High-income Asia Pacific (81.3 g/day), Oceania (43.3 g/day), Tropical Latin America (39.7 g/day), Western Europe, (34.9 g/day), East Asia (34.2 g/day)	Tropical Latin America (91.3 g/day), Southern Latin America (80.0 g/day), Australasia (75.9 g/day), Eastern Europe (64.1 g/day), Andean Latin America (60.0 g/day)	Central Latin America (44.4 g/day), high-income North America (34.6 g/day), Central Europe (32.2 g/day), Eastern Europe (31.8 g/day), Andean Latin America (27.0 g/day)
Regions with lowest levels (mean consumption)	South Asia (27.6 g/day), East Asia (42.3 g/day), Southern Sub-Saharan Africa (48.6 g/day), Central Asia (64.6 g/day), East Sub-Saharan Africa (68.5 g/day)	Central Asia (86.1 g/day), Oceania (102.7 g/day), high-income North America (123.3 g/day), Southern Latin America (123.3 g/day), Caribbean (139.7 g/day)	Southern Sub-Saharan Africa (0.3 g/day), Southern Latin America (0.6 g/day), Tropical Latin America (2.3 g/day), high-income Asia Pacific (2.6 g/day), Central Sub-Saharan Africa (3.0 g/day)	High-income Asia Pacific (9.4 g/day), East Asia (11.1 g/day), Tropical Latin America (13.7 g/day), Central Europe (15.4 g/day), South Asia (16.0 g/day)	Southern Sub-Saharan Africa (9.2 g/day), Central Latin America (10.4 g/day), Central Asia (11.9 g/day), Central Europe (15.9 g/day), South Asia (17.2 g/day)	South Asia (7.3 g/day), Southeast Asia (26.0 g/day), West Sub-Saharan Africa (33.0 g/day), East Sub-Saharan Africa (34.1 g/day), Caribbean (34.4 g/day)	East Asia (4.1 g/day), North Africa/Middle East (4.4 g/day), West Sub-Saharan Africa (6.0 g/day), East Sub-Saharan Africa (6.4 g/day), high-income Asia Pacific (7.0 g/day)
Regions with greater statistical uncertainty	Andean Latin America,* West Sub-Saharan Africa,† Caribbean†	Andean Latin America,* Caribbean,† Asia Central†‡	Andean Latin America,* Oceania,†‡ Central Sub-Saharan Africa†‡	South Asia,‡ Eastern Europe,* Oceania†‡	Andean Latin America,* Oceania,†‡ South Asia‡	Oceania,†‡ Central Sub-Saharan Africa,†‡ Andean Latin America,* Caribbean†	South Asia,‡ Oceania,†‡ Central Sub-Saharan Africa,†‡ Andean Latin America*
Range across 187 countries (overall variation)	19.2–325.1 g/day (17-fold)	34.6–493.1 g/day (14.3-fold)	0.2–152.7 g/day (1000-fold)	1.3–334.3 g/day (255-fold)	6.0–87.6 g/day (14.5-fold)	3.0–124.2 g/day (41.5-fold)	2.5–66.1 g/day (27-fold)
Countries with highest levels (mean consumption)	Jamaica (325.1 g/day), Malaysia (301.1 g/day), Jordan, (275.6 g/day), Greece (255.3 g/day), New Zealand (251.7 g/day), Barbados (239.3 g/day)	Zimbabwe (493.1 g/day), Botswana (475.9 g/day), Swaziland (451.9 g/day), Greece (426.0 g/day), Laos (369.8 g/day), Samoa (344.6 g/day)	Maldives (152.7 g/day), Cambodia (92.3 g/day), Malaysia (85.6 g/day), Myanmar (82.3 g/day), Laos (54.4 g/day), Vietnam (51.0 g/day)	Seychelles (334.3 g/day), Malaysia (285.2 g/day), Chad (246.3 g/day), Mauritius (238.6 g/day), Indonesia (238.5 g/day), Mali (196.2 g/day)	Japan (87.6 g/day), Maldives (67.6 g/day), South Korea (66.6 g/day), Portugal (64.7 g/day), Spain (64.6 g/day), Iceland (58.4 g/day), Denmark (58.1 g/day), Norway (57.3 g/day)	Central African Republic (124.2 g/day), Gabon (108.4 g/day), Samoa (107.9 g/day), Sweden (100.8 g/day), Algeria (100.7 g/day), Paraguay (98.4 g/day), United Arab Emirates (96.3 g/day)	Panama (66.1 g/day), other Central Latin American nations (44.6–56.4 g/day), Poland (48.8 g/day), Latvia (43.6 g/day), Belarus (41.5 g/day), Mexico (40.5 g/day)
Countries with lowest levels (mean consumption)	Ethiopia (19.2 g/day), Nepal (19.9 g/day), India (22.7 g/day), Vanuatu (30.0 g/day), Pakistan (31.6 g/day)	Vanuatu (34.6 g/day), Philippines (45.9 g/day), Hungary (61.9 g/day), Switzerland (65.1 g/day), Armenia (66.4 g/day), Georgia (68.7 g/day)	Lesotho (0.2 g/day), other Southern Sub-Saharan African nations (0.2–0.4 g/day), Argentina (0.6 g/day), Uruguay (0.6 g/day), Chile (0.7 g/day), Iceland (0.9 g/day)	Hungary (1.3 g/day), Croatia (2.6 g/day), Albania (2.9 g/day), Turkey (3.1 g/day), Macedonia FYROM (3.3 g/day), Pakistan (3.4 g/day)	Zimbabwe (6.0 g/day), Guatemala (6.3 g/day), Honduras (7.3 g/day), Nicaragua (8.1 g/day), occupied Palestinian Territory (8.2 g/day), Mongolia (8.3 g/day)	India (3.0 g/day), Sri Lanka (11.6 g/day), Maldives (13.0 g/day), Bhutan (13.8 g/day), Indonesia (13.9 g/day), Comoros (15.7 g/day)	Occupied Palestinian Territory (2.5 g/day), other North Africa/Middle Eastern nations (2.6–3.8 g/day), Comoros (2.9 g/day), Afghanistan (3.7 g/day), North Korea (3.8 g/day)
Western Europe mean consumption (95% uncertainty interval)	165.2 g/day (155.0–175.6)	171.3 g/day (165.0–178.0)	3.5 g/day (3.3–3.8 g/day)	61.8 ay (55.9–68.0)	34.9 g/day (32.4–37.6)	59.9 g/day (57.3–62.8)	26.4 g/day (24.8–28.2)
Western Europe range with country examples	93.8 g/day in the UK and 99.4 g/day in Ireland to 209.8 g/day in Israel and 255.3 g/day in Greece	65.1 g/day in Switzerland and 76.4 g/day in Iceland to 275.3 g/day in Cyprus and 426.0 g/day in Greece	0.9 g/day in Iceland and 1.5 g/day in Ireland to 8.2 g/day in the Netherlands and 8.5 g/day in Israel	11.9 g/day in Belgium and Italy to 92.2 g/day in Iceland and 130.1 g/day in Germany	15.0 g/day in the Netherlands and 18.4 g/day in Germany to 64.6 g/day in Spain and 64.7 g/day in Portugal	32.9 g/day in Israel and 34.6 g/day in France to 90.6 g/day in Cyprus and 100.8 g/day in Sweden	4.7 g/day in Greece and 7.2 g/day in Israel to 37.2 g/day in Finland and 38.5 g/day in Austria
US mean consumption (95% uncertainty interval)	93.2 g/day (86.0–100.2)	115.6 g/day (110.3–120.6)	4.5 g/day (4.2–4.9)	47.3 g/day (43.7–50.9)	20.1 g/day (18.9–21.5)	44.9 g/day (42.7–47.5)	35.7 g/day (32.9–38.9)
Number of countries achieving optimal mean intakes, corresponding adult global population (% of global adult population)	≥300 g/day: 2 of 187 countries, 19 million people (0.4%)	≥400 g/day: 4 of 187 countries, 17 million people (0.4%)	≥4 oz (28.35 g)/week: 26 of 187 countries, 422 million people (9.6%)	≥2.5 (50 g) servings/day: 23 of 187 countries, 335 million people (7.6%)	≥3.5 (100 g) servings/week: 12 of 187 countries, 193 million people (4.4%) ≥2.0 (100 g) servings/week: 73 of 187 countries, 2.1 billion people (47.5%)	≤1 (100 g) serving/week: 5 of 187 countries, 900 million people (20.3%)	0 intake: 0 of 187 countries, 0 people (0%) ≤1 (50 g) serving/week: 55 of 187 countries, 1.7 billion people (38.5%)
Number of countries not achieving optimal mean intakes, corresponding adult global population (% of global adult population)	<300 g/day: 185 of 187 countries, 4.4 billion people (99.6%)	<400 g/day: 183 of 187 countries, 4.4 billion people (99.6%)	<4 oz (28.35 g)/week: 161 of 187 countries, 4 billion people (90.4%)	<2.5 (50 g) servings/day: 164 of 187 countries, 4.1 billion people (92.4%)	<3.5 (100 g) servings/week: 175 of 187 countries, 4.2 billion people (95.6%) <2.0 (100 g) servings/week: 114 of 187 countries, 2.3 billion people (52.5%)	>1 (100 g) serving/week: 182 of 187 countries, 3.5 billion people (79.7%)	Other than 0 intake: 187 of 187 countries, 4.42 billion people (100%) >1 (50 g) serving/week: 132 of 187 countries, 2.72 billion people (61.5%)

*Owing to higher within-country statistical uncertainty in the raw data.

†Owing to limited country-specific raw data on consumption levels.

‡Owing to greater variation in consumption levels between countries in the region.

Worldwide, mean vegetable consumption (including legumes) was 208.8 g/day (table 3 and figure 2), with less variation (approximately 3.4-fold) compared with fruits across highest (294.4 g/day) to lowest (86.1 g/day) regions (figure 5). Among individual nations, intake ranged from 34.6 to 493.1 g/day: highest in Zimbabwe, other South Sub-Saharan African nations (Botswana and Swaziland) and Greece; and lowest in Vanuatu, the Philippines, Hungary, Switzerland, Armenia and Georgia (see eTable 3). High vegetable intake was primarily due to high consumption of legumes in Southern Sub-Saharan Africa and Tropical Latin America; due to both legumes and other vegetables in Central and Eastern Sub-Saharan Africa; and due to vegetables alone in Greece, high-income Asia Pacific and East Asia. Only 4 of 187 countries had intake levels ≥400 g/day, representing 17 million adults and 0.4% of the world adult population.

Across regions and countries, intakes of fruits and vegetables were generally strongly intercorrelated: regional r(Spearman)=0.8, national r=0.7. Yet, key exceptions were seen. For example, in Sub-Saharan Africa, Eastern Asia and Southern Asia, consumption of vegetables was considerably higher than of fruits. This discordance was most notable in Zimbabwe (vegetables 493.1 vs fruits 32.2 g/day), Botswana (475.9 vs 38.2 g/day), Ethiopia (195.1 vs 19.2 g/day), Nepal (201.8 vs 19.9 g/day), China (293.1 vs 39.4 g/day) and India (160.0 vs 22.7 g/day). Similarly, in certain nations, fruit intake was much higher than vegetables, for instance, in Caribbean nations such as Jamaica (vegetables 165.2 vs fruits 325.1 g/day), Antigua and Barbuda (118.4 vs 203.9 g/day), Bahamas (132.2 vs 210.5 g/day) and Saint Lucia (120.5 vs 189.1 g/day); and in Switzerland (65.1 vs 194.1 g/day), Philippines (45.9 vs 110.6 g/day) and Malaysia (142.7 vs 301.1 g/day).

**Figure 3:**
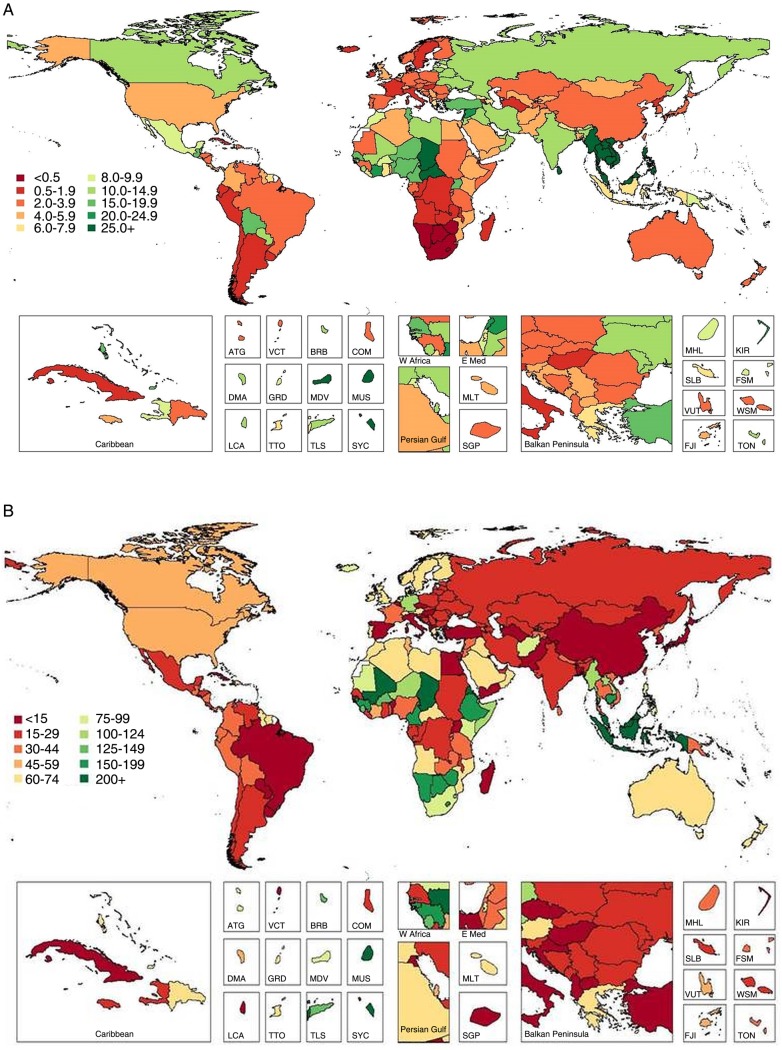
Global and regional mean nut and seed (A) and whole grain (B) intake (g/d) in 2010 for adults ≥20 years of age (see eTable 3 for numerical mean estimates and uncertainty intervals).

**Figure 5:**
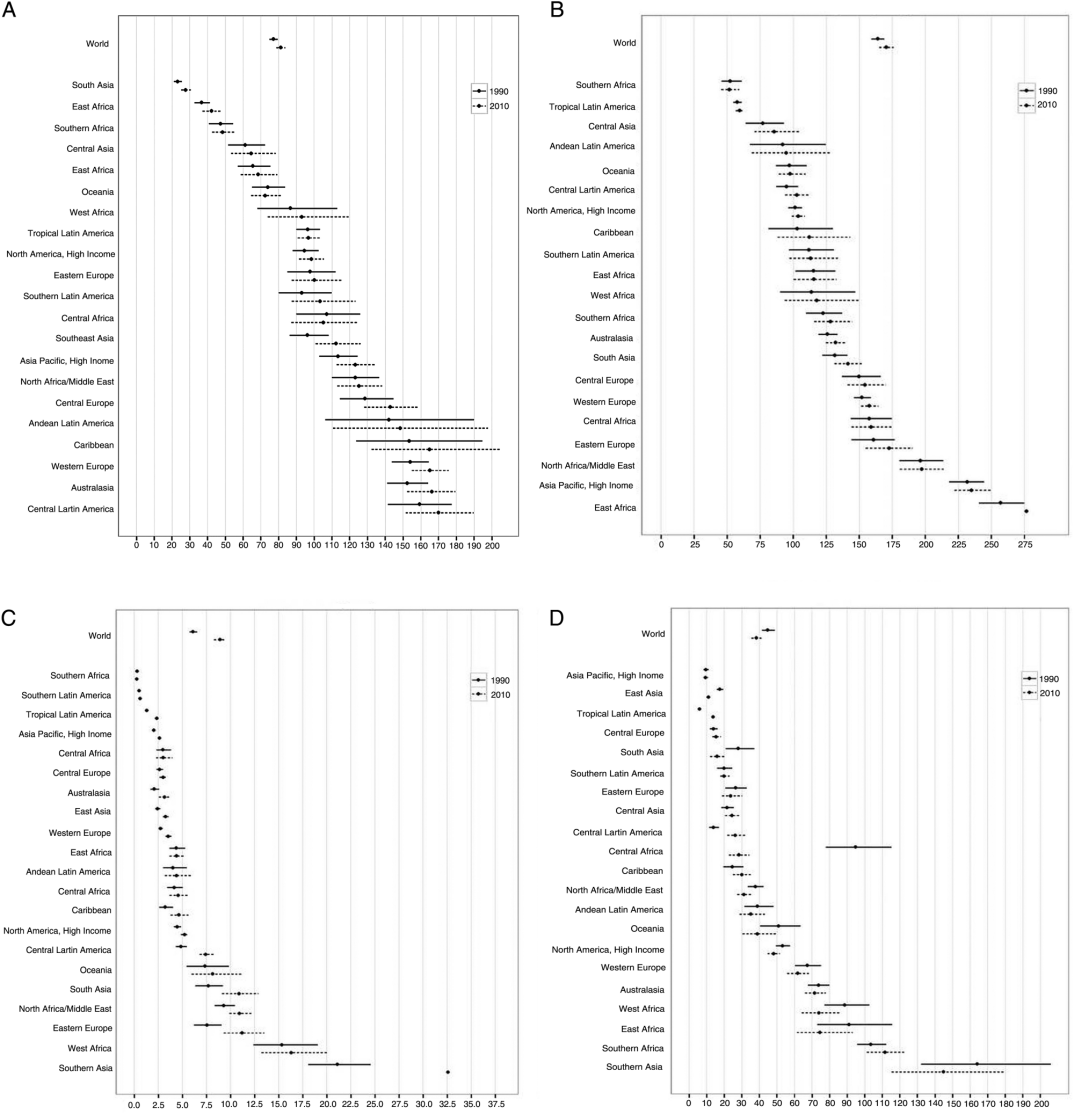
Global and regional mean fruit (A), vegetable (B), nut and seed (C), and whole grain (D) intake in 1990 and 2010, for adults ≥20 years of age in relation to their uncertainty.

Mean global intake of nuts/seeds was 8.9 g/day (95% UI 8.3–9.5), with tremendous variation by region (>100-fold; from 0.3 to 32.6 g/day; table 3 and figures 3 and 5) and nation (roughly 1000-fold; from 0.2 to 152.7 g/day; see eTable 3). Maldives had highest consumption, followed by Southeast Asian nations (Cambodia, Malaysia, Myanmar, Laos, Vietnam). Lowest intakes were identified in South Sub-Saharan African nations (eg, Lesotho and Namibia), Argentina, Uruguay and Iceland (see eTable 3). A total of 26 countries had mean consumption ≥4.1 oz (28.35) servings/week, consistent with optimal levels, representing 420 million adults and 9.6% of the global adult population. Intakes varied considerably within geographic areas, with, for example, very low intakes in certain Southern and Eastern Sub-Saharan African nations but substantially higher intakes in others (eg, the Seychelles 38.4 g/day, Mauritius 27.6 g/day) as well as in Western Sub-Saharan Africa (eg, up to 28.1 g/day in Chad). Similarly, in the Mediterranean basin, intakes ranged from 1.7–3.9 g/day in Italy, Spain and Algeria to 16.5–23.5 g/day in Tunisia, Turkey and Lebanon.

**Figure 6:**
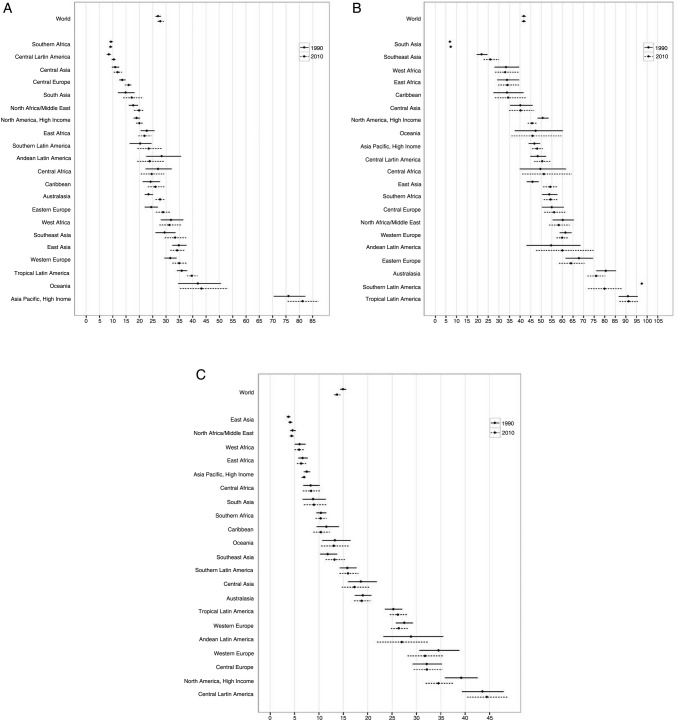
Global and regional mean seafood (A), unprocessed red meat (B), and processed meat (C) intake (g/d) in 1990 and 2010, for adults ≥20 years of age in relation to their uncertainty.

Globally, mean consumption of whole grains was 38.4 g/day (table 3 and figure 3). Across GBD regions, 15-fold differences (from 71.6 to 144.9 g/day) were identified (figure 5); and across countries, even greater variation (from 1.3 to 334.3 g/day). Seychelles, other Sub-Saharan African nations such as Chad and Mauritius, and Southeast Asian nations such as Malaysia and Indonesia, had highest intakes (see eTable 3). Lowest intakes were in Hungary; other Central European nations such as Albania, Croatia and Turkey; and South Asian nations such as Pakistan and Bangladesh. Overall, 23 of 187 countries had mean whole grain intake ≥2.5 (50 g) servings/day, representing 335 million adults and 7.6% of the world adult population. Both regionally and nationally, consumption of whole grains did not correlate strongly with consumption of nuts (r=0.30 and 0.12, respectively). Of interest, in several Southern Sub-Saharan African nations and Nordic nations, intakes of whole grains were in the highest range while intakes of nuts/seeds were in the lowest range. Several countries in Southeast Asia had high intakes of both, including Malaysia, Cambodia, Maldives and Myanmar.

Global mean seafood intake was 27.9 g/day (95% UI 26.9–29.1). As might be expected, highest intakes were identified in island nations including Japan, Maldives and Iceland; as well as in South Korea, Portugal, Spain, Denmark and Norway (see eTable 3). Lowest intakes were in Zimbabwe, Central Latin American nations (eg, Guatemala and Honduras), the Occupied Palestinian Territory and Mongolia. Notably, 73 of 187 countries had mean seafood intakes ≥2.0 (100 g) servings/week, representing 2.1 billion adults and 47.5% of the world adult population. Conversely, only 12 of 187 countries had mean intakes ≥3.5 (100 g) servings/week, representing 193 million adults and 4.4% of the global adult population. Across world regions, mean intakes varied 9-fold (from 9.2 to 81.3 g/day; table 3 and figures 4 and 5); and across nations, 14.5-fold (from 6.0 to 87.6 g/day). Interestingly, seafood intake did not correlate strongly with consumption of either unprocessed red meat (regional r=0.12; national r=0.02) or processed meat (regional r=−0.20; national r=−0.04).

**Figure 4:**
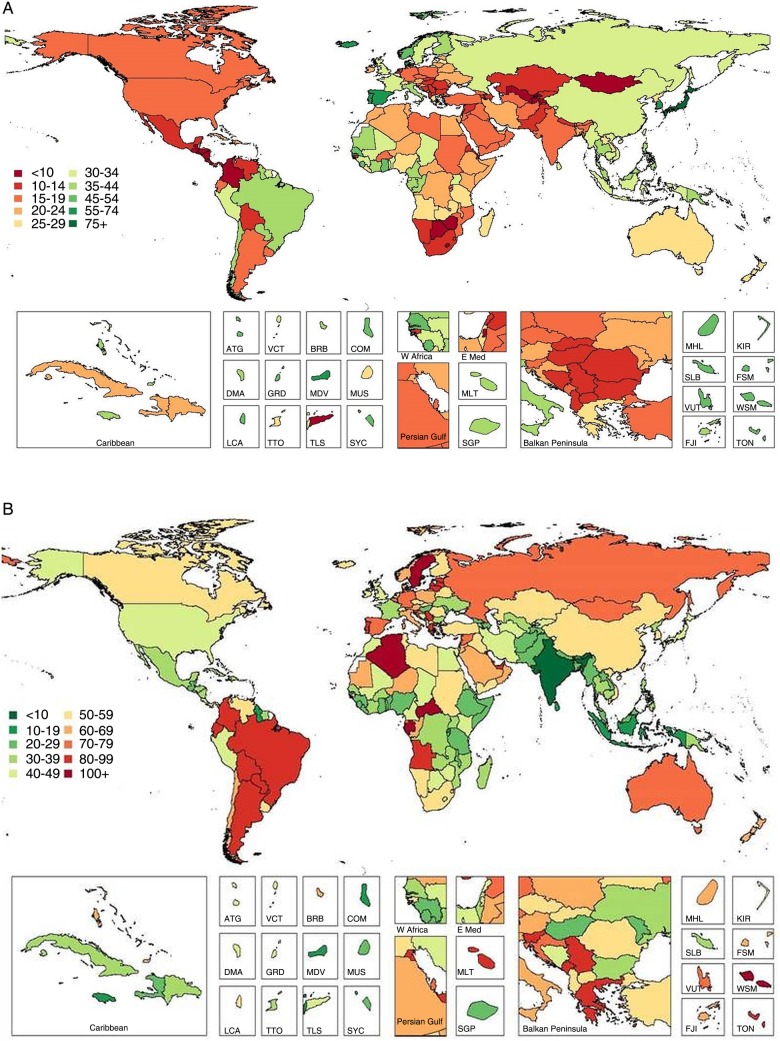
Global and regional mean seafood (A), unprocessed red meat (B) and processed meat (C) intake (g/d) in 2010 for adults ≥20 years of age (see eTable 3 for numerical mean estimates and uncertainty intervals).

**Figure 4:**
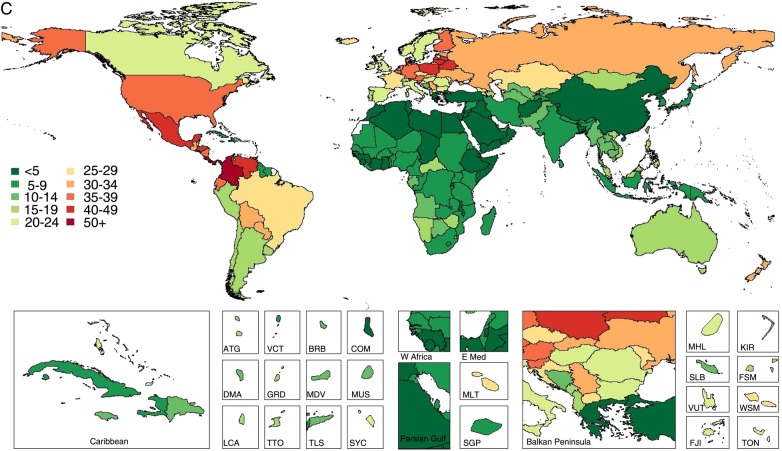
(Continued)

Mean consumption of unprocessed red meat (beef, pork or lamb) was 41.8 g/day (95% UI 40.8–42.8), with a 12.5-fold range across regions (table 3 and figures 4–6). By country, intake ranged from 3.0 to 124.2 g/day (see eTable 3). Highest intakes were seen in Central African Republic, Gabon, Samoa, Sweden, Algeria, Paraguay and United Arab Emirates. Lowest intakes were identified in South and Southeast Asian nations such as India, Sri Lanka, Maldives, Bhutan and Indonesia, as well as in Comoros. Overall, only 5 of 187 countries had intakes ≤1 (100 g) serving/week, representing 900 million adults and 20.3% of the global adult population.

Compared with unprocessed red meat, mean global intake of processed meat was much lower at 13.7 g/day (95% UI 13.2–14.3; table 3 and figures 4–6). Notably, both regionally and nationally, intakes of processed meat did not correlate strongly with unprocessed red meat (r=0.10 and 0.35, respectively). Certain countries had higher intakes of both (eg, Latin American nations such as Colombia, Paraguay and Bolivia; Eastern and Western European nations such as Lithuania, Latvia, Estonia, Poland, Malta, Germany, Belgium, Austria and Russia); and others, lower intakes of both (eg, Asian nations such as India, Sri Lanka, Malaysia, Bangladesh, Pakistan and Singapore; and Sub-Saharan nations such as Rwanda, Sierra Leone and Benin; see eTable 3). In contrast, several countries had higher intakes of unprocessed red meat and lower intakes of processed meat (eg, North African/Middle Eastern nations such as Algeria, Jordan and Libya; Greece; China) and others, lower intakes of unprocessed red meat and higher intakes of processed meat (eg, other Central Latin American nations such as El Salvador, Nicaragua, Guatemala and Mexico; and Moldova and Ukraine in Eastern Europe). There were no countries with zero (optimal) consumption, although 55 of 187 countries had intakes ≤1 (50 g) serving/week, representing 1.7 billion adults and 38.5% of the global adult population.

### Differences in consumption by sex and age

Within both regions and countries, the energy-adjusted consumption of each food was not substantially different in women versus men, although women nearly always had slightly greater consumption of more healthful foods and lower intake of less healthful foods (see eFigures 1–5 and eTables 4–5 and 8–11). For example, compared with men, women generally consumed slightly more fruits (+21.7 g/day) and vegetables (+15.9 g/day); and less processed meats (−3.1 g/day).

Similarities and differences were seen in intakes across age groups (see eFigure 6). For example, whereas intakes of nuts/seeds were relatively similar by age, fruit consumption varied more greatly by age, with generally higher intakes among older adults, particularly in Western European, the Caribbean and high-income Asia Pacific nations. Vegetable consumption varied similarly by age, especially in high-income Asia Pacific, East Asian and North African/Middle Eastern nations. Whole grain consumption also increased at higher ages, mainly in South and East Sub-Saharan Africa and Southeast Asia. The positive age association was most profound for seafood intake; this was especially evident in high-income Asia Pacific and Oceania. In contrast, an inverse age association was identified for intakes of both unprocessed red meat and processed meat. The former was most apparent in Tropical and South Latin America and Eastern Europe; and the latter, in Central Latin America, Central and Eastern Europe, and high-income North America.

### Changes in consumption between 1990 and 2010

Between 1990 and 2010, mean global fruit intake increased by +5.3 g/day (95% UI 2.1–8.6; figure 5). By region, intake nominally increased in 18 of 21 regions, although these increases only achieved statistical significance in South Asia (+3.8 g/day (0.6–7.4); see eTable 6). Among South Asian countries, the only statistically significant increase occurred in India (+3.8 g/day (0.6–7.4)); in all other South Asian countries, intakes increased non-significantly, with the exception of Afghanistan, where intakes decreased non-significantly. Intake decreased in three regions, although none of these decreases were statistically significant, including in Central Sub-Saharan Africa (−1.6 g/day (−26.9 to 24.2)) and Tropical Latin America (−0.8 g/day (−9.8 to 7.9)). Among countries, in addition to India, statistically significant increases occurred in Bosnia and Herzegovina (+16.7 g/day (0.7–34.0)), and South Korea (+14.4 g/day (2.4–26.0)). No countries had statistically certain decreases; largest non-significant decreases were seen in North Korea (−26.5 (−81.3 to 24.0)), Iraq (−19.9 (−97.6 to 55.0)) and Lebanon (−17.6 (−65.5 to 28.7)).

Across these two decades, mean worldwide vegetable intake was stable (global change +1.8 g/day (−5.6 to 9.2); figure 5). Non-significant increases were seen in Eastern Europe (+9.5 g/day (−14.7 to 32.3)) and Southern (+8.2 g/day (−5.7 to 22.5)), Southeastern (+7.9 g/day (−13.8 to 28.0)), Eastern (+6.5 g/day (−17.6 to 30.2)) and Central (+5.8 g/day (−16.7 to 29.3)) Asia. Intake decreased in Tropical Latin America (−27.5 g/day (−47.6 to −9.0)). By nation, significant increases were seen in Sri Lanka (+34.2 g/day (6.5–60.2); see eTable 6); and largest decreases in Mozambique (−40.9 g/day (−65.2 to −17.2)) and Brazil (−27.8 g/day (−47.9 to −10.2)).

Worldwide, nut and seed consumption increased by +2.3 g/day (1.6–3.1; figure 5), including significant increases in 13 regions and non-significant trends towards increases in 6 more. Largest increases were in Southeast Asia (+11.0 g/day (5.3–17.3)), Eastern Europe (+3.7 g/day (1.3–6.3)) and South Asia (+3.2 (1.0–5.5)). Intake remained stable in Southern and Eastern Sub-Saharan Africa. Among individual nations, largest increases occurred in Vietnam (+32.7 g/day (14.5–55.4)), the Philippines (+13.9 g/day (3.6–25.1)), Belize (13.9 g/day (7.6–22.2)), Dominica (+9.0 g/day (4.0–15.2)), Saint Lucia (+8.5 g/day (3.6–14.6)) and Paraguay (+7.6 g/day (3.7–12.5); see eTable 6). Greatest decreases were in Guinea-Bissau (−19.0 g/day (−34.9 to −6.4)) and Burkina Faso (−5.6 g/day (−9.4 to −2.2)).

In contrast to global increases in intakes of fruits and nuts/seeds, and stable intake of vegetables, the global intake of whole grains decreased between 1990 and 2010, by −8.5 g/day (−13.9 to −4.2; figure 5). We identified largest decreases in Central Sub-Saharan Africa (−65.9 g/day (−87.1 to −47.0)), South Asia (−12.1 g/day (−22.7 to −4.1), North Africa/the Middle East (−8.1 g/day (−13.9 to −1.7)) and East Asia (−6.8 g/day (−9.0 to −4.7); see eTable 6). Two regions had statistically significant increases: Central (+12.1 g/day (6.7–18.7)) and Tropical (+7.6 g/day (6.6–8.6)) Latin America. Nationally, greatest absolute decreases were in Congo (−372.1 g/day (−558.8 to −236.9)), Maldives (−237.1 g/day (−373.2 to −131.3)), Gabon (−163.3 g/day (−234.9 to −109.4)), Angola (−148.7 g/day (−232.6 to −83.8)) and Djibouti (−137.8 g/day (−226.5 to −66.7)). The largest national increases were in Mauritius (+161.2 g/day (91.9–255.4)), Cape Verde (+150.2 g/day (100.9–220.8)), Zimbabwe (+107.2 g/day (62.7–161.6)), Sierra Leone (+86.6 g/day (49.2–130.9)) and Laos (+75.3 g/day (44.1–109.6)).

Global seafood intake remained stable between 1990 and 2010 (+1.0 g/day (−0.5 to 2.5; figure 6). Slight increases were identified in five regions, the largest in Central (+4.2 g/day (0.4–8.1)), Eastern (+3.8 g/day (1.4–6.1)) and Southeastern (+2.8 g/day (0.0–5.5)) Asia (see eTable 7); and slight, non-significant decreases in Western (−4.8 g/day (−13.2 to 2.9)), Southern (−2.3 g/day (−9.0 to 4.6)) and Eastern (−2.0 g/day (−6.0 to 2.0)) Sub-Saharan Africa. By nation, mean seafood intakes were also relatively unchanged. We identified largest increases in South Korea (+10.4 g/day (2.9–18.2)), Croatia (+10.4 g/day (5.2–15.7)), Moldova (+8.9 g/day (2.9–15.5)), Belarus (+7.9 g/day (1.4–15.0)) and Jamaica (+6.6 g/day (0.3–13.1)); and significant decreases only in North Korea (−22.6 g/day (−38.9 to −9.1)), Guinea-Bissau (−9.2 g/day (−17.0 to −2.5)) and Taiwan (−7.3 g/day (−12.0 to −3.2)).

Mean unprocessed red meat consumption increased marginally between 1990 and 2010, by 1.5 g/day (0.2–2.9), while mean processed meat intake was stable (−0.1 g/day (−0.9 to 0.8); figure 6). In only one region, East Asia, unprocessed red meat intake significantly increased (+8.3 g/day (4.2–12.5)). In contrast, significant decreases were evident in Southern Latin America (−16.7 g/day (−28.8 to −5.2)) and high-income North America (−5.0 g/day (−8.2 to −1.6); see eTable 7). Across countries, greatest increases were in Latvia (+15.2 g/day (1.6–30.2)), South Korea (+13.4 g/day (8.8–17.8)), Mozambique (+9.0 g/day (6.7–11.3)) and China (+8.6 g/day (4.5–12.7)). During this same period, Uruguay experienced the largest declines in unprocessed red meat intake (−63.3 g/day (−115.8 to −19.0)), followed by Argentina (−20.7 g/day (−27.6 to −14.3)), Bulgaria (−9.9 g/day (−16.2 to −3.6)), Canada (−7.1 g/day (−11.1 to −2.9)), the Netherlands (−6.1 g/day (−11.9 to −0.6)) and the USA (−4.7 g/day (−8.2 to −1.2)). No regions or countries had statistically significant increases or decreases in processed meat intake between 1990 and 2010.

## Discussion

### Relevance of findings for public health

By 2030, the global economic burden due to cardiometabolic diseases and other NCDs is estimated to reach $47 trillion.^49^ National and international organisations have highlighted the critical need to reduce health and economic burdens of NCDs, much of which occur prematurely and can be prevented or delayed.^1^ ^49^ ^50^ In recent years, multiple diet–disease relationships have been established, in particular for fruits and vegetables, whole grains, nuts/seeds, seafood, and unprocessed and processed meats.^51^ Overall, suboptimal diet is now the single leading preventable cause of NCDs,^2^ making food-based research a top priority for public health.

This systematic investigation provides, for the first time, to our knowledge, quantitative estimates based on individual-level dietary assessments of global intakes of key foods influencing chronic diseases, including by region, country, age and sex; as well as uncertainty in these estimates. These global data identify key challenges and opportunities for assessing and optimising diets. Our findings also facilitate quantitative assessment of disease burdens attributable to these dietary factors, for example, using state-transition Markov models,^52^ ^53^ food impact models^43^ ^54^ ^55^ and comparative risk assessment.^2^ ^3^ ^6^ ^56^ ^57^ Understanding global patterns and impact of suboptimal diet is essential to develop priorities for prevention initiatives and public policies to reduce disease burdens and disparities around the world. Thus, these findings are highly relevant for the global scientific community, health professionals, policymakers, advocacy groups and the public.

### Principal findings and interpretation

Worldwide, mean intakes of healthful foods including fruits, vegetables, nuts/seeds, whole grains and fish were substantially below current recommendations or optimal intakes. Increasing fruits and vegetables, historically targeted in combination, has been a public health goal for many nations. Our findings suggest that fruit intake has modestly increased globally over the past two decades, while vegetable intake has remained constant. Yet, in 2010, the great majority of countries in the world had mean intakes below optimal, indicating a need for further efforts to increase production, reduce spoilage and reduce cross-national disparities.^58^ Policy approaches to increase fruit and vegetable intake must also consider ways to address cost, which for the poorest populations represents a major barrier to consumption. Our results also demonstrate, for the first time, to our knowledge, the disparity between fruit versus vegetable consumption in certain nations, such as China, highlighting the need to assess and target their intakes separately.

Intakes of whole grains and nuts/seeds, which until recently have received less public health attention than fruits and vegetables, were similarly far below optimal in most nations. Yet, our findings demonstrate substantial heterogeneity. For example, certain Asian nations had high intakes of both, while others (eg, Japan and China) had low intakes of both, perhaps partly attributable to high intakes of refined rice in the latter. Several Sub-Saharan African nations had high intakes of whole grains, likely representing amaranth grain, millet, teff, sorghum and fonio, but very low intakes of nuts/seeds. Interestingly, our findings suggest that global consumption of nuts/seeds increased, while global consumption of whole grains decreased, over the past two decades. Largest increases in nut/seeds were identified in Southeast and South Asia, while largest decreases in whole grains were seen in Sub-Saharan Africa and East and South Asia; the latter may be due to increasing use of refined starches (eg, starchy vegetables and white rice) replacing traditional whole grains.

Consistent with historical cultures and local availability, highest seafood intake was evident in Pacific island nations, the Mediterranean Basin, South Korea and Japan. In the latter two nations, seafood is often consumed in salted form, which may partly explain their high rates of stroke and gastric cancer.^27^ ^29^ ^59^ ^60^ Additionally, in some countries such as Maldives, Malaysia, Barbados, Seychelles, Iceland and Denmark, consumption of long-chain omega-3′ is substantially higher than in other nations with similar overall seafood intake,^24^ suggesting the importance of both overall seafood consumption and the usual types of fish consumed (eg, oily fish and white fish). We identified extremely low seafood consumption (generally <15 g/day) in Central Latin America, Sub-Saharan Africa and several (non-Mediterranean) nations in North Africa/Middle East. Overall, our findings demonstrate the lack of adequate seafood intake in most of the world, and a lack of improvement globally between 1990 and 2010, highlighting the importance of increasing sustainable aquaculture and fishing practices, as well as global distribution and policy efforts to increase consumption.

We identified relatively independent consumption levels of unprocessed red meat versus processed meat across nations. Growing evidence suggests that processed meats are particularly adverse for cardiometabolic health, perhaps attributable to their far higher levels of preservatives (especially sodium).^40^ ^61^ Interestingly, over the past two decades, global processed meat consumption was stable, while unprocessed red meat intake slightly increased. The latter likely reflects the cultural and economic prioritisation of meat consumption in low-income and middle-income countries, which also provides an increasing source of calories, iron, zinc and protein among the poorest populations of the world. These increases over time must be balanced against adverse environmental effects,^62^ particularly in comparison to other protein sources such as poultry, eggs and dairy.^63^

Interestingly, energy-adjusted intakes of each food were relatively similar by sex, although women tended to have marginally higher intakes of more healthful foods and lower intakes of less healthful foods. Reported intakes are standardised to the same isocaloric intake (2000 kcal/day) across genders and age groups, and thus represent compositional and not absolute intakes, which generally appear most relevant for health and disease risk.^41^ In comparison, we identified stronger age differences for nearly all foods, with more healthful intake levels at older ages. Whether these identified age patterns represent changes in diets with ageing versus birth cohort effects (ie, individuals born in earlier decades having different dietary patterns) cannot be distinguished on the basis of these data. The latter is particularly concerning, as sustained poor diets into older ages will lead to further increases in global chronic diseases over time.

### Strengths

We performed systematic searches and extensive direct contacts that allowed us to identify, assess and compile global individual-level dietary intake data, largely from national studies, on multiple key foods worldwide, including by region, nation, age, sex and time. To ensure measurement comparability across surveys, identified surveys were evaluated for eligibility, measurement comparability and representativeness; and consistency across surveys was maximised by standardised data extraction and analyses, reinforcing validity and generalisability. Metrics and measurement units were standardised across surveys and were based on the evidence for effects on chronic disease risk. We characterised optimal consumption levels for each key food, placing observed consumption levels in context and enabling consideration of potential impacts on disease burdens in a consistent and comparable manner across countries. Recommended intakes may differ in selected populations, such as children, pregnant and lactating women, or diseased populations. Intakes were adjusted for total energy, accounting for differences in body size, metabolic efficiency and physical activity, and reducing measurement error (under-reporting and over-reporting);^43^ and sensitivity analyses without energy adjustment were similar. We developed a Bayesian hierarchical imputation model to account for differences in intakes versus availability, and to address representativeness and comparability, and related effects on sampling and modelling uncertainty.

### Limitations

Despite comprehensive approaches to data identification and retrieval, individual-level intake data were limited for certain foods, countries and time periods; in particular, more intake data were available in 2010 than in 1990, and relatively few intake data were available in most Sub-Saharan African nations, and on nuts/seeds and whole grains. FAO data have inherent limitations, these being mainly that not only official but also unofficial and estimated or imputed data have been used, particularly for Sub-Saharan African countries, and that such data often overestimate individual-based dietary intakes.^46^ Furthermore, several combinations of foods in the FAO data were possible, and future investigations can further expand on potential combinations. Our investigation takes advantage of FAO food balance sheet data in a multilevel model that allows them to provide additional information across all countries and years, but also lets them be appropriately adjusted to account for their error and variation based on relationships with multiple individual dietary surveys in countries having both. Our systematic evaluation of representative surveys from around the world also provides a roadmap of gaps and global priorities for further individual-based dietary assessments. We focused on foods with probable or convincing evidence for impact on chronic diseases; and many other foods (eg, poultry, eggs, refined grains) were not included. We also focused on adults; and corresponding global individual-level intakes in children would be of great interest. We evaluated intakes based on age, sex, country and time; and other subnational factors such as education and rural/urban status could also influence intakes. Thus, our findings represent the best available, yet still imperfect, data on global intakes of key foods. In ongoing work, we are updating our searches, data collection and modelling, to overcome each of these prior limitations (http://www.globaldietarydatabase.org; anticipated results in 2018).

### Conclusions and policy implications

Optimising dietary habits to improve population health requires systematically identified and evaluated data. Despite the wealth of evidence on health effects of dietary habits, the patterns and distributions of consumption of major foods around the world have been surprisingly understudied. Our findings provide comparable, consistent data on intakes of key foods on a global scale, formally incorporating methodological heterogeneity and sampling and modelling uncertainty, and elucidating differences by age and sex. These data can be used to assess consumption within and across nations and regions; investigate correlates and drivers of dietary intakes and nutrition transitions over time; estimate corresponding disease burdens attributable to suboptimal intakes; and model, design and implement specific dietary policies to reduce disease in and disparities between different nations. National priorities and policies for reducing diet-related illness must address barriers to optimal consumption,^64^ such as production, distribution, cost, sustainability, local cultures and food practices, potential industry opposition, equity assurance and political feasibility.
